# Evaluation of cultural awareness and intercultural sensitivity among nursing students: a comparison of Türkiye and Somalia

**DOI:** 10.1186/s12912-026-04811-9

**Published:** 2026-05-23

**Authors:** Şeyma Zehra Altunkürek, Esra Yıldız, Efe Gençler, Salmo Abdi Mohamed Ali

**Affiliations:** 1 Department of Nursing, Somalia Mogadishu Recep Tayyip Erdoğan Faculty of Health Sciences, University of Health Sciences, Mogadishu, Somalia; 2 Public Health Nursing Department, Faculty of Nursing, Atatürk University, Erzurum, Türkiye; 3 Public Health Nursing Department, Gülhane Faculty of Nursing, University of Health Sciences, Ankara, Türkiye

**Keywords:** Cultural awareness, Intercultural sensitivity, Transcultural nursing, Somalia, Türkiye

## Abstract

**Background:**

Cultural awareness and intercultural sensitivity are essential competencies for nursing students providing care in multicultural settings. This study aimed to compare cultural awareness and intercultural sensitivity among nursing students in Türkiye and Somalia, and to examine the sociodemographic factors associated with these outcomes.

**Methods:**

This cross-sectional comparative study included nursing students from Somalia (*n* = 214) and Türkiye (*n* = 199). Data were collected using a self-report questionnaire, the Intercultural Sensitivity Scale (ISS), and the Cultural Awareness Scale (CAS). Chi-square tests, independent samples t-tests, and simple and multiple linear regression analyses were used.

**Results:**

Turkish students had significantly higher CAS total scores than Somali students (167.84 ± 25.38 vs. 151.00 ± 23.87), and significantly higher scores on all CAS subdimensions. ISS total scores were also significantly higher among Turkish students (87.78 ± 12.66 vs. 77.12 ± 9.69), and Turkish students scored significantly higher on all ISS subdimensions. In regression analyses, nationality remained a significant predictor of both cultural awareness and intercultural sensitivity. Providing care to individuals from different cultural backgrounds was an additional positive predictor of cultural awareness.

**Conclusions:**

Nursing students in Türkiye demonstrated higher levels of cultural awareness and intercultural sensitivity compared to those in Somalia. Although nationality was significantly associated with both outcomes, this relationship may reflect broader educational, clinical, and sociocultural differences between the two countries. Despite reporting greater intercultural care experience, Somali students had lower scores on both scales. These findings suggest that exposure to cultural diversity alone may be insufficient and highlight the importance of structured educational approaches incorporated into the curriculum for the development of cultural competence in nursing education.

**Trial registration:**

Clinical trial number: not applicable.

## Background

Culture encompasses the values, beliefs, and practices that individuals transmit from one generation to the next through social interaction [1]. This structure, which directly shapes health perceptions, illness experiences, and care processes, has fundamentally changed the nature of health services as a result of dynamics such as globalization, international workforce mobility, wars, and forced migration [1, 2]. In particular, Türkiye’s geopolitical position and its proximity to surrounding conflict zones make the country an important transit and settlement point for intense migration movements, thereby increasing cultural diversity and mobility [3, 4]. Health professionals interact more frequently with individuals who have different languages, religions, values, and health beliefs, making it essential to consider not only the biomedical but also the cultural dimensions of care [5, 6]. For this reason, cultural awareness and intercultural sensitivity stand out among the key factors that determine the quality of care. These two concepts are regarded as essential competencies, particularly for the delivery of high quality care in health services.

According to the Campinha-Bacote model, cultural competence is defined as a dynamic process that includes the dimensions of cultural awareness, knowledge, skill, encounters, and desire, whereas the Purnell model addresses this competence as a multidimensional structure at the individual, family, and community levels [7, 8]. These theoretical approaches indicate that cultural awareness and intercultural sensitivity are core components of the broader construct of cultural competence [9, 10]. Cultural awareness is defined as the process by which individuals recognize their own cultural values, biases, and assumptions and become open to different cultural perspectives, whereas intercultural sensitivity refers to the capacity to demonstrate appropriate, respectful, and effective attitudes and behaviors in interactions with people from different cultures [10, 11]. These two concepts represent complementary cognitive, affective, and behavioral dimensions. The literature emphasizes that possessing cultural knowledge alone is not sufficient to provide culturally sensitive care unless it is supported by appropriate communication skills and attitudes [12, 13]. Therefore, addressing cultural awareness and intercultural sensitivity together is of critical importance for a holistic evaluation of nursing students’ competencies in these areas [9, 10].

The development of cultural awareness and intercultural sensitivity is not limited to individual characteristics, but is also closely associated with the educational environment and learning experiences [14–16]. Indeed, recent studies have shown that the content of nursing education programs, pedagogical approaches, and opportunities for clinical practice play an important role in the development of these skills [14, 15]. In addition, educational strategies such as simulation based learning, intercultural interaction experiences, and reflective learning approaches have been shown to improve not only students’ level of knowledge, but also their levels of cultural sensitivity and awareness [16, 17].

However, nursing education curricula show important systematic and structural differences between countries in terms of developing these competencies [14, 18, 19]. A global review of nursing education shows that elements such as curriculum structure, clinical practice standards, accreditation systems, and educational philosophy vary considerably across countries, and that countries’ levels of economic development and health policies directly shape the focus of the curriculum [19, 20]. In particular, nursing education models in North America and Oceania place the concepts of cultural competence and cultural safety at the center of the curriculum and adopt an approach grounded in critical thinking and self-reflection [9, 21]. In these systems, students are encouraged not only to recognize cultural differences, but also to analyze the cultural context within the framework of issues such as power relations, inequalities, and health equity [21, 22]. In contrast, nursing education systems in Europe have been shaped by efforts toward standardization through the Bologna Process, with educational programs being developed mainly around transcultural nursing models [14, 18]. By contrast, in many Asian and developing countries, nursing education primarily focuses on technical and biomedical skills, while issues related to cultural competence are generally addressed in a limited, implicit, or ethical context [5, 23]. It has been reported that this situation may limit the systematic development of students’ cultural interaction skills, and that the development of cultural competence and sensitivity may not reach a sufficient level in the absence of structured educational interventions [15].

Accordingly, it has been reported that the structural differences observed in nursing education approaches across countries play an important role in the development of students’ levels of cultural awareness and intercultural sensitivity [14, 15, 20]. It is emphasized that these concepts are shaped not only by individual characteristics, but also by structural elements such as the content of educational programs, teaching methods, and learning environments [14, 15]. Therefore, a comparative examination of these differences between educational systems is considered essential for identifying the key structural determinants that influence nursing students’ levels of cultural awareness and intercultural sensitivity [14, 20].

Although some studies in the literature have addressed cultural awareness and intercultural sensitivity together, research conducted among nursing students in Türkiye has shown that these two concepts are influenced by various individual and experiential factors, such as gender, foreign language proficiency, interaction with different cultures, participation in exchange programs, and willingly choosing the profession [24–26]. Similarly, studies conducted in different countries have shown that nursing students’ levels of cultural awareness and intercultural sensitivity are associated with variables such as educational experiences, international interactions, and intercultural contact [11, 15]. In particular, studies conducted in Europe and North America emphasize that intercultural education programs and structured learning experiences play an important role in the development of these concepts [14, 20]. However, a review of the existing literature shows that studies have largely focused on Western centered samples, and that research directly comparing countries representing different sociocultural structures remains limited [14, 15]. In particular, the lack of comparative studies conducted between countries that share similar cultural and religious characteristics but have different educational systems is noteworthy [15, 20]. This situation has led to the limited identification of the educational and sociocultural determinants of cultural awareness and intercultural sensitivity [14, 27].

The aim of this study was to compare the levels of cultural awareness and intercultural sensitivity among nursing students studying in Türkiye and Somalia, and to examine the individual and educational factors associated with these differences. The findings of the study are expected to contribute to the development of more culturally inclusive nursing education programs, curriculum development processes aligned with international standards, and the strengthening of patient safety in multicultural care settings. The research questions of this study are as follows:

1. What are the levels of cultural awareness and intercultural sensitivity among nursing students in Türkiye and Somalia?
2. Is there a significant difference in the levels of cultural awareness and intercultural sensitivity between nursing students in Türkiye and Somalia?
3. Which sociodemographic and related variables are significant predictors of cultural awareness and intercultural sensitivity levels?

## Methods

### Study design

This study was conducted using a cross-sectional, comparative, and descriptive research design to compare the levels of cultural awareness and intercultural sensitivity among nursing students studying in Türkiye and Somalia, and to determine the sociodemographic and related variables associated with these levels.

### Study population and sample size

The study population consisted of nursing students enrolled at a university in the city of Erzurum, Türkiye, and a university in the city of Mogadishu, Somalia. Both institutions provide undergraduate-level nursing education and represent diverse socio-cultural groups. Participants were selected using a non-probability convenience sampling method. The inclusion criteria were: (1) voluntary participation, (2) provision of verbal and written informed consent, (3) being a nursing student and (4) being literate in Turkish. An a priori power analysis was conducted using G*Power (version 3.1) for independent samples t-test to determine the minimum required sample size for group comparisons. Assuming a two-tailed test, a significance level of 0.05, a statistical power of 0.80, and a small-to-medium effect size (Cohen’s d ≈ 0.30), the required sample size was calculated as approximately 176 participants per group (total *N* ≈ 352). To enhance the robustness and generalizability of the findings and to account for potential missing or incomplete data, a larger sample was targeted. Ultimately, a total of 413 participants were included in the final analysis, exceeding the minimum required sample size.

### Measurements

The researchers collected sociodemographic data using a structured questionnaire that assessed the following characteristics: gender, age, class level, marital status, income level, foreign language proficiency, desire to work abroad, knowledge of intercultural nursing, having friends from different countries, having been abroad, providing care to individuals from different cultures, perceived adequacy while providing such care, experiencing difficulties while providing such care, having taken an intercultural nursing course, and preference for including an intercultural nursing course in the curriculum.

#### The Cultural Awareness Scale (CAS)

The Cultural Awareness Scale (CAS) was used to assess nursing students’ fundamental knowledge, skills, attitudes, strengths, and limitations related to providing culturally congruent nursing care. The instrument was developed by Rew et al. [28] and later adapted into Turkish by Başalan İz and Bayık Temel, who confirmed its validity and reliability. The CAS consists of 36 items rated on a 7 point Likert scale ranging from 1 (Strongly Disagree) to 7 (Strongly Agree), and includes several reverse scored items (8, 9, 12, 16, 19, 22, and 36). The Turkish version of the scale comprises four subdimensions: General Educational Experience, Cognitive Awareness, Behaviors and Interaction, and Classroom/Clinical Practices and Research. Higher total scores indicate greater cultural awareness. The internal consistency of the original scale was reported as α = 0.82 [28], while the Turkish adaptation demonstrated a Cronbach’s alpha of α = 0.89 [29]. In the present study, the CAS showed very high internal consistency in both Turkish (α = 0.95) and Somali (α = 0.93) samples. Subscale reliability coefficients were also high, ranging from 0.85 to 0.92 for Turkish participants and from 0.82 to 0.90 for Somali participants.

#### The Intercultural Sensitivity Scale (ISS)

The Intercultural Sensitivity Scale (ISS), originally created by Chen and Starosta [11], was employed to measure nursing students’ intercultural sensitivity levels. Its Turkish version was adapted, validated, and assessed for reliability by Bulduk et al. [30]. The ISS includes 24 items distributed across five dimensions: interaction engagement, respect for cultural differences, interaction confidence, interaction enjoyment, and interaction attentiveness. Items are scored on a five-point Likert scale from 1 (Strongly Disagree) to 5 (Strongly Agree). Total scores range between 24 and 120, where higher values reflect increased intercultural sensitivity. In the original development study, the scale’s Cronbach’s alpha was reported as 0.88 [11]; in contrast, the Turkish adaptation yielded α = 0.72 in Bulduk et al. [30], whereas in the present study, the ISS demonstrated high internal consistency for both Turkish (α = 0.93) and Somali (α = 0.91) samples. Subscale reliability coefficients ranged from 0.81 to 0.89 for Turkish participants and from 0.80 to 0.87 for Somali participants.

Permission to use the Turkish versions of the Cultural Awareness Scale and the Intercultural Sensitivity Scale was obtained from the relevant authors of the scales before data collection. Both scales were used in accordance with the permissions granted and any applicable conditions of use.

### Data collection

Data were collected between January and March 2025. In both countries, the researchers at the respective universities followed the same standardized data collection procedures. Classroom visits were conducted on regular school days, during which students were informed about the study’s aims and procedures. All participants were provided with comprehensive information about the study’s purpose, procedures, and confidentiality safeguards. They were explicitly notified that their involvement was completely voluntary, that they could discontinue participation at any point without needing to offer a reason, and that choosing to participate or not would in no way influence their academic standing. The Somali participants were students enrolled at a Turkish affiliated university in Mogadishu and were proficient in Turkish, which allowed the data collection process to be conducted using the same Turkish language instruments. After receiving full information, students who agreed to participate provided verbal and written informed consent, and the questionnaires were subsequently distributed for self-administration. Completion of the forms took approximately 25 min.

### Data analysis

IBM SPSS Statistics 26.0 was used to analyze the data. Statistical significance was set at *p* < 0.05. Normality was assessed using skewness and kurtosis values. Frequency, percentage, mean, and standard deviation were used for descriptive analyses. Chi-square tests were used to compare categorical variables. Independent samples t tests were used for group comparisons, and Cohen’s d effect sizes were calculated. Simple and multiple linear regression analyses were also performed.

### Ethical considerations

This study received approval from the Atatürk University Non-Interventional Clinical Research Ethics Committee (Decision No: B.30.2.ATA.0.01.00/33, Date: 31 January 2025) and from the Mogadishu Somalia Turkey Recep Tayyip Erdoğan Training and Research Hospital Ethics Committee (Decision No: MSTH/20529, 1160, Date: 22 December 2024). Additionally, written authorization to conduct the research was obtained from the administrations of the nursing departments at both institutions. All students were informed about the purpose and procedures of the study, and verbal and written informed consent was obtained from those who agreed to participate.

## Results

**Table 1:** Distribution and comparison of students’ sociodemographic characteristics

Students’ Sociodemographic Characteristics	Türkiye (*n* = 199)		Somalia (*n* = 214)		Total (*n* = 413)		Test
	*n*	%	*n*	%	*n*	%	
**Age**							
18–20	91	45.7	118	55.1	209	50.6	*X*^2^=3.654 *p* = 0.06
21+	108	54.3	96	44.9	204	49.4	
**Gender**							
Male	58	29.1	11	5.1	69	16.7	*X*^2^=42.7 ***p*** < 0.001**
Female	141	70.9	203	94.9	344	83.3	
**Class Level**							
1st Year	59	29.6	59	27.6	118	28.6	*X*^2^=3.263 *p* = 0.35
2nd Year	61	30.7	62	29.0	123	29.8	
3rd Year	36	18.1	54	25.2	90	21.8	
4th Year	43	21.6	39	18.2	82	19.9	
**Marital Status**							
Married	3	1.5	20	9.3	23	5.6	*X*^2^=12.047 ***p*** < 0.001**
Single	196	98.5	194	90.7	390	94.4	
**Income Level**							
Low	64	32.2	41	19.2	105	25.4	*X*^2^=44.033 ***p*** < 0.001**
Medium	118	59.3	97	45.3	215	52.1	
High	17	8.5	76	35.5	93	22.5	
**Knowledge of a Foreign Language**							
Yes	90	45.2	189	88.3	279	67.6	*X*^2^=87.356 ***p*** < 0.001**
No	109	54.8	25	11.7	134	32.4	
**Desire to Work Abroad**							
Yes	125	62.8	161	75.2	286	69.2	*X*^2^=7.469 ***p*** = 0.006*
No	74	37.2	53	24.8	127	30.8	
**Knowledge of Intercultural Nursing**							
Yes	72	36.2	126	58.9	198	47.9	*X*^2^=23.009 ***p*** < 0.001**
No	49	24.6	42	19.6	91	22.0	
Partially	78	39.2	46	21.5	124	30.0	
**Having Friends from Different Countries**							
Many	25	12.6	58	27.1	83	20.1	*X*^2^=14.304 ***p*** < 0.001**
Few	107	53.8	103	48.1	210	50.8	
None	67	33.7	53	24.8	120	29.1	
**Having Been Abroad**							
Yes	34	17.1	64	29.9	98	23.7	*X*^2^=9.366 ***p*** = 0.002**
No	165	82.9	150	70.1	315	76.3	
**Providing Care to Individuals from Different Cultures**							
Yes	58	29.1	106	49.5	164	39.7	*X*^2^=17.901 ***p*** < 0.001**
No	141	70.9	108	50.5	249	60.3	
**Perceived Adequacy in Providing Care to Individuals From Different Cultures**							
Competent	12	6	76	35.5	88	21.3	*X*^2^=54.557 ***p*** < 0.001**
Partially Competent	131	65.8	89	41.6	220	53.3	
Not Competent	56	28.1	49	22.9	105	25.4	
**Experiencing Difficulties While Providing Care to Individuals from Different Cultures**							
Yes	56	28.1	48	22.4	104	25.2	*X*^2^=1.785 *p* = 0.19
No	143	71.9	166	77.6	309	74.8	
**Having Taken an Intercultural Nursing Course**							
Yes	58	29.1	0	0	58	14	*X*^2^=72.562 ***p*** < 0.001**
No	141	70.9	214	100	355	86	
**Preference for Including an Intercultural Nursing Course in the Curriculum**							
Yes	146	73.4	170	79.4	316	76.5	*X*^2^=2.116 *p* = 0.14
No	53	26.6	44	20.6	97	23.5	

*X*^2^: Chi-Square Test; *p < 0.05; **p < 0.005

As shown in Table 1, statistically significant differences were found in the variables of gender, marital status, income level, knowledge of a foreign language, desire to work abroad, knowledge of intercultural nursing, having friends from different countries, having been abroad, providing care to individuals from different cultures, perceived adequacy while providing such care, and having taken an intercultural nursing course (*p* < 0.05). Students in Somalia had statistically significantly higher proportions of being female and married, having a high income level, knowing a foreign language, wanting to work abroad, having knowledge of intercultural nursing, having many friends from different countries, having been abroad, providing care to individuals from different cultures, and perceiving themselves as adequate while providing such care, compared with students in Türkiye (*p* < 0.05). In contrast, the proportion of students who had taken an intercultural nursing course was higher in Türkiye than in Somalia (*p* < 0.05) (Table 1).

**Table 2:** Comparison of students’ cultural awareness and intercultural sensitivity scores by nationality

Scale	Türkiye (*n* = 199)	Somalia (*n* = 214)	t	df	*p*	d
	x̄±SD	x̄±SD				
**Cultural Awareness Scale (CAS)**						
**Total**	167.84 ± 25.38	151.00 ± 23.87	6.950	411	< 0.001**	0.684
General Educational Experience	30.37 ± 7.60	28.32 ± 8.14	2.637	411	0.009*	0.260
Behaviors and Interaction	36.70 ± 5.70	33.36 ± 5.73	5.929	411	< 0.001**	0.584
Cognitive Awareness	33.10 ± 5.38	29.80 ± 6.61	5.520	411	< 0.001**	0.544
Classroom/Clinical Practices and Research	67.67 ± 12.84	59.50 ± 13.17	6.368	411	< 0.001**	0.627
**Intercultural Sensitivity Scale (ISS)**						
**Total**	87.78 ± 12.66	77.12 ± 9.69	9.646	411	< 0.001**	0.950
Interaction Engagement	25.94 ± 4.37	22.38 ± 4.53	8.097	411	< 0.001**	0.798
Respect for Cultural Differences	22.19 ± 4.16	18.84 ± 3.93	8.417	411	< 0.001**	0.828
Interaction Confidence	17.78 ± 2.87	16.94 ± 3.62	2.595	411	0.009*	0.256
Interaction Enjoyment	10.91 ± 2.68	9.69 ± 2.64	4.682	411	< 0.001**	0.458
Interaction Attentiveness	10.96 ± 2.11	9.28 ± 2.59	7.194	411	< 0.001**	0.707

x̄: Mean, SD: Standard Deviation, t: Independent samples t test, df: Degrees of Freedom, d: Cohen’s Effect Size, *p < 0.05; **p < 0.005

As shown in Table 2, students in Türkiye had significantly higher scores than those in Somalia in terms of both total scores and all subdimensions (*p* < 0.05). The total cultural awareness score was significantly higher among students in Türkiye (167.84 ± 25.38) than among students in Somalia (151.00 ± 23.87) (t = 6.950, *p* < 0.001), and this difference had a medium effect size (d = 0.68). A medium effect size was observed for the subdimensions of behaviors and interaction (d = 0.58), cognitive awareness (d = 0.54), and classroom/clinical practices and research (d = 0.63), whereas the effect size was small for the general educational experience subdimension (d = 0.26). The total intercultural sensitivity score was also significantly higher among students in Türkiye (87.78 ± 12.66) than among students in Somalia (77.12 ± 9.69) (t = 9.646, *p* < 0.001), and the effect size of this difference was large (d = 0.95). Large effect sizes were found for the subdimensions of interaction engagement (d = 0.80), respect for cultural differences (d = 0.83), and interaction attentiveness (d = 0.71), whereas a medium effect size was observed for interaction enjoyment (d = 0.46) and a small effect size for interaction confidence (d = 0.26). Nationality was associated with statistically significant differences in both cultural awareness and intercultural sensitivity (Table 2).

**Table 3:** Multiple regression models for cultural awareness and intercultural sensitivity

Independent Variable	CAS (Model 1) B (SE)	β	*p*	CAS (Model 2) B (SE)	β	*p*	ISS (Model 1) B (SE)	β	*p*	ISS (Model 2) B (SE)	β	*p*
Constant	167.849 (1.745)	–	< 0.001	160.293 (10.401)	–	< 0.001	87.784 (0.796)	–	< 0.001	83.089 (4.761)	–	< 0.001
Nationality, Somalia	**-16.845 (2.424)**	**-0.324**	**< 0.001**	**-20.901 (3.267)**	**-0.402**	**< 0.001**	**-10.662 (1.105)**	**-0.430**	**< 0.001**	**-12.092 (1.495)**	**-0.487**	**< 0.001**
Age	–	–	–	0.324 (0.513)	0.032	0.528	–	–	–	0.254 (0.235)	0.053	0.280
Gender, Male	–	–	–	1.024 (3.502)	0.015	0.770	–	–	–	-0.443 (1.603)	-0.013	0.782
Class level	–	–	–	0.374 (1.231)	0.016	0.762	–	–	–	0.727 (0.564)	0.064	0.198
Income level, Low	–	–	–	-5.107 (2.942)	-0.086	0.083	–	–	–	-1.455 (1.347)	-0.051	0.281
Income level, High	–	–	–	3.037 (3.210)	0.049	0.345	–	–	–	0.345 (1.469)	0.012	0.814
Knowledge of a foreign language, Yes	–	–	–	-0.504 (2.920)	-0.009	0.863	–	–	–	-0.250 (1.337)	-0.009	0.852
Having been abroad, Yes	–	–	–	0.380 (2.982)	0.006	0.899	–	–	–	0.864 (1.365)	0.030	0.527
Having friends from different countries, Few	–	–	–	1.861 (2.878)	0.036	0.518	–	–	–	-2.080 (1.317)	-0.084	0.115
Having friends from different countries, Many	–	–	–	1.825 (3.772)	0.028	0.629	–	–	–	-2.172 (1.727)	-0.070	0.209
Having taken an intercultural nursing course, Yes	–	–	–	-5.681 (3.947)	-0.076	0.151	–	–	–	-2.907 (1.807)	-0.081	0.108
Providing care to individuals from different cultures, Yes	**–**	**–**	**–**	**5.456 (2.694)**	**0.103**	**0.044**	**–**	–	–	1.629 (1.233)	0.064	0.187

B: Unstandardized regression coefficient; SE: Standard error; β: Standardized regression coefficient

### Reference categories

Nationality=Türkiye; Gender=Female; Income level=Middle; Knowledge of a foreign language = No; Having been abroad = No; Having friends from different countries=None; Having taken an intercultural nursing course = No; Providing care to individuals from different cultures = No.

### Cultural Awareness Scale (CAS)

Model 1: *R* = 0.324, R²=0.103, F = 48.309, *p* < 0.001

Model 2: *R* = 0.369, R²=0.111, F = 5.266, *p* < 0.001

### Intercultural Sensitivity Scale (ISS)

Model 1: *R* = 0.430, R²=0.183, F = 93.037, *p* < 0.001

Model 2: *R* = 0.455, R²=0.184, F = 8.725, *p* < 0.001

According to the regression analysis presented in Table 3, nationality was a significant predictor in the simple regression model established for cultural awareness (β=-0.324, *p* < 0.001; R²=0.103), indicating that Somali students had lower scores. This variable remained significant in the multiple model as well (β=-0.402, *p* < 0.001; R²=0.111). In this model, only the variable of providing care to individuals from different cultures was found to be significant and positive (β = 0.103, *p* = 0.044). For intercultural sensitivity, nationality was also a significant predictor (β=-0.430, *p* < 0.001; R²=0.183), and it remained significant in the multiple model (β=-0.487, *p* < 0.001; R²=0.184). None of the other variables made a significant contribution (*p* > 0.05). Overall, nationality remained the most consistent variable retaining statistical significance in both models, whereas the contribution of the other variables was limited (Table 3).

## Discussion

This study aimed to comparatively examine the levels of cultural awareness and intercultural sensitivity among nursing students studying in Türkiye and Somalia, and to identify the sociodemographic and related variables that significantly predicted these levels. The study found that the mean total scores for cultural awareness and intercultural sensitivity were significantly higher among students in the Türkiye sample than among those in the Somalia sample (*p* < 0.05). In this respect, the study offers an original contribution to the literature, where cultural awareness and intercultural sensitivity have mostly been examined through single country samples or comparisons involving Western countries, by comparing nursing students in Türkiye and Somalia within the same study.

Students in Somalia were found to have higher rates of knowledge of a foreign language, desire to work abroad, and having been abroad than students in Türkiye. Similar to our findings, a study conducted with nursing and medical students in South Africa reported a marked tendency to go abroad after graduation, while another study conducted in Ghana found that most nursing students planned to migrate after completing their education [31, 32]. These studies suggest that international mobility and migration tendency may be important issues among some health sciences students in Africa. In a study conducted by Koç et al. with nursing students, characteristics such as knowledge of a foreign language, desire to study in another country, and desire to work abroad were reported to be associated with intercultural sensitivity [25]. That study suggested that knowledge of a foreign language and desire to work abroad may be characteristics to consider in relation to intercultural sensitivity. This result may be related to the greater orientation of students in Somalia toward international education and career opportunities. In addition, the widespread use of English and the desire to access better working conditions in the health field may have increased students’ orientation toward studying or working abroad.

In our study, students in Somalia were found to have higher rates of having friends from different countries, providing care to individuals from different cultures, and perceiving themselves as adequate while providing such care than students in Türkiye. In the study conducted by Muslu and Tuzcu with nursing students, it was reported that the experience of providing care to individuals from different cultures may be associated with cultural awareness [33]. Similarly, studies conducted with nursing and health sciences students have indicated that encountering or interacting with individuals from different cultures may be associated with intercultural sensitivity [24, 26]. These studies report that experiences such as providing care to individuals from different cultures and engaging in intercultural interaction are variables that may be considered in relation to cultural awareness and intercultural sensitivity. In our study, the fact that students in Somalia reported more social and clinical contact experience with individuals from different cultures may be related to their presence in a social and care environment that is more open to intercultural interaction. However, since the quality and duration of intercultural experiences and the extent to which these experiences were integrated into educational processes were not evaluated, how such contacts are reflected in students’ cultural development should be interpreted with caution.

Some students in Türkiye were found to have taken an intercultural nursing course, whereas students in Somalia had not taken this course. However, the proportion of students who reported having knowledge of intercultural nursing was higher in the Somalia sample than in the Türkiye sample. In addition, the proportion of students who wanted an intercultural nursing course to be included in the curriculum was high in both groups, and no significant difference was found between the groups (*p* > 0.05). In a study conducted by Tosun and Sinan in 2020 with nursing students in Türkiye, only one third of the students were reported to have received intercultural nursing education [34]. In another study conducted by Halabi and de Beer in 2018, approximately half of the nursing students reported that they wanted to take an intercultural nursing course [35]. These results indicate that interest in intercultural nursing was high in both samples. The fact that students in the Somalia sample more frequently reported having knowledge of intercultural nursing suggests that this difference may be related not only to education received through formal courses, but also to students’ social environments and intercultural encounter experiences.

In our study, the mean total score of the Cultural Awareness Scale (CAS) was 167.84 ± 25.38 among students in Türkiye and 151.00 ± 23.87 among students in Somalia. Cultural awareness was found to be significantly higher among students in Türkiye, and this difference had a medium effect size (*p* < 0.05). Studies conducted with nursing students in Türkiye have reported CAS mean scores ranging from 168 to 172 [33, 36]. However, in the international literature, the mean total CAS score was reported as 194 among nursing students in Slovenia and 181 among Vietnamese nursing students [37, 38]. In the multicenter study conducted by Sagarra-Romero et al. in 2024, the mean total CAS score was found to be 190 in the Spanish sample, 185 in the Belgian sample, 201 in the Portuguese sample, and 182 in the Türkiye sample. A significant difference was found among these countries in terms of total CAS scores, and the Türkiye sample was found to have significantly lower scores than the other European country samples [39]. These findings indicate that the Türkiye sample in our study was similar to national studies in terms of the CAS total score, but remained below the scores reported in some European and Asian samples. It is noteworthy that although indicators of international orientation and social and clinical intercultural contact were higher in the Somalia sample, the CAS total score was lower. This finding may suggest that cultural awareness cannot be explained solely by individual experiences such as contact with different cultures, and that how students make sense of these experiences in terms of the care process and cultural differences may also be important. In addition, providing intercultural nursing education in a structured manner may contribute to students’ ability to transform these experiences into cultural awareness.

When the CAS subdimensions were examined, students in Türkiye were found to have higher scores than students in Somalia across all subdimensions (*p* < 0.05). When effect sizes were evaluated by subdimension, a small difference was found in the General Educational Experience subdimension, whereas medium differences were observed in the Behaviors and Interaction, Cognitive Awareness, and Classroom/Clinical Practices and Research subdimensions. The multicenter study conducted with nursing students in Spain, Türkiye, Belgium, and Portugal also reported that CAS subdimensions could show different results across country samples [39]. These findings indicate that differences between countries in cultural awareness are not limited only to the total score, but may vary according to the areas represented by the subdimensions. In our study, the smallest difference being observed in the General Educational Experience subdimension suggests that the divergence between the two samples was more limited in general perceptions related to the educational environment. In contrast, the medium differences observed in the Behaviors and Interaction, Cognitive Awareness, and Classroom/Clinical Practices and Research subdimensions suggest that the greater intercultural contact reported in the Somalia sample may not have been reflected to the same extent in students’ behaviors related to cultural awareness, their ways of evaluating cultural differences, and their clinical learning experiences. Therefore, not only the presence or frequency of intercultural contact, but also how this contact is addressed in the educational process, how it is supported in clinical practice, and how it is interpreted by students may be important in explaining the differences in CAS subdimensions.

The mean total score of the Intercultural Sensitivity Scale (ISS) was 87.78 ± 12.66 among students in Türkiye and 77.12 ± 9.69 among students in Somalia. When students’ levels of intercultural sensitivity were compared in our study, students in Türkiye were found to have higher scores than students in Somalia, and this difference had a large effect size (*p* < 0.05). Studies examining the intercultural sensitivity levels of nursing students in Türkiye have reported mean ISS scores ranging from 85 to 92 [2, 25, 36, 40–42]. In a study comparing students in Türkiye and the United Kingdom, the mean total ISS score was reported to be higher in the United Kingdom sample, while another study comparing samples from the United States and Türkiye reported that students in the United States had higher mean ISS scores [43, 44]. These results suggest that intercultural sensitivity may be related not only to students’ individual experiences, but also to countries’ nursing education approaches, communication based learning processes, the visibility of intercultural care in the curriculum, and the characteristics of clinical learning environments.

Students in Türkiye were found to have higher mean scores than students in Somalia across all ISS subdimensions (*p* < 0.05). Large effect sizes were observed in the Interaction Engagement, Respect for Cultural Differences, and Interaction Attentiveness subdimensions, whereas a medium effect size was found in the Interaction Enjoyment subdimension and a small effect size in the Interaction Confidence subdimension. When studies in the literature are examined, ISS subdimensions have been reported to show different results across country samples, and the Türkiye sample was reported in some studies to have lower scores in certain subdimensions compared with the United Kingdom, the United States, and some European countries [39, 43, 44]. Studies conducted with nursing students in Türkiye have also reported that some ISS subdimensions may be associated with attitudes toward different cultural groups and with taking an intercultural nursing course [45, 46]. In our study, the more pronounced differences observed particularly in the Interaction Engagement, Respect for Cultural Differences, and Interaction Attentiveness subdimensions may suggest that the communication based and attitudinal dimensions of intercultural sensitivity may be more strongly influenced by educational and sociocultural processes. In contrast, the more limited difference in the Interaction Confidence subdimension suggests that the divergence between the two samples remained lower in this subdimension.

In the multiple regression analysis, nationality was significantly associated with both CAS and ISS scores, whereas experience in providing care to individuals from different cultural backgrounds showed a limited association only with cultural awareness (*p* < 0.05). Similar associations have also been reported in comparative studies conducted across different cultural and educational settings [39, 43, 44]. Kaya et al. also reported that ethnocentrism and moral sensitivity were significant predictors of intercultural sensitivity among nursing students [47]. However, the observed relationship with nationality should not be interpreted as a direct or independent causal effect. Nationality may reflect broader contextual factors, such as differences in educational systems, clinical learning environments, sociocultural structures, and opportunities for intercultural interaction. Accordingly, the findings suggest that cultural awareness and intercultural sensitivity are shaped not only by individual characteristics but also by processes that depend on educational, clinical, and sociocultural contexts. Furthermore, the limited association observed for intercultural care experience may indicate that exposure to different cultures alone is insufficient, and that the quality, structure, and educational integration of such experiences may play a more important role in the development of outcomes related to cultural competence.

## Limitations

Several limitations should be considered when interpreting the findings of this study. First, the use of self-reported measures may introduce response bias, and the cross-sectional design limits causal inference. Second, although some variables were included, not all potential confounding factors (e.g., gender, socioeconomic status, prior intercultural experience) could be fully controlled. Third, despite a similar educational framework and shared language of instruction, underlying socio-cultural differences between the groups may have influenced the findings. In addition, although the Somali participants were proficient in Turkish and studied in a Turkish-affiliated university where Turkish was the language of instruction, the CAS and ISS instruments used in this study had not undergone formal cultural adaptation or validity and reliability testing specifically within the Somali context. Therefore, potential cultural and linguistic differences may have affected measurement equivalence and the interpretation of the findings. Finally, the study was conducted in a single institution in each country, which may limit the generalizability of the findings. Therefore, the results should be interpreted with caution.

## Conclusions and recommendation

The findings of this study demonstrated that nursing students in Türkiye had higher levels of cultural awareness and intercultural sensitivity compared to nursing students in Somalia. Despite reporting greater intercultural care experience, Somali students had lower CAS and ISS scores. Although nationality remained significantly associated with cultural awareness and intercultural sensitivity, this relationship may reflect broader educational, clinical, and sociocultural differences between the two countries rather than an independent explanatory effect. These findings suggest that exposure to cultural diversity alone may be insufficient for the development of cultural awareness and intercultural sensitivity. Accordingly, structured educational approaches incorporated into the curriculum may contribute to strengthening cultural competence in nursing education. In addition, further multicenter and comparative studies directly examining educational and social factors are needed to better understand the determinants of these competencies.

## Data Availability

The datasets generated and/or analyzed during the current study are not publicly available due to ethical and confidentiality considerations but are available from the corresponding author on reasonable request.

## Abbreviations

CAS: Cultural awareness scale
ISS: Intercultural sensitivity scale
SPSS: Statistical package for the social sciences
